# Catalysing Environmental Action: a Governance Framework for Enhancing Individual Participation in Sub-Saharan Africa’s Plastic Circular Economy

**DOI:** 10.1007/s00267-024-02044-7

**Published:** 2024-09-17

**Authors:** Ambisisi Ambituuni, Olubunmi Ajala, Patrick Schroeder, Muyiwa Oyinlola

**Affiliations:** 1 Department of Management, Birmingham Business School, University of Birmingham, Birmingham, UK; 2 School of Economics, Finance and Accounting, Faculty of Business and Law, Coventry University, Coventry, UK; 3 Chatham House, London, UK; 4 School of Engineering and Sustainable Development, Faculty of Computing, Engineering and Media, De Montfort University, Leicester, UK

**Keywords:** Circular economy, Plastic pollution, Action recipe, Public governance, Sub-Saharan Africa, Individual action

## Abstract

Plastic waste poses a significant challenge to achieving sustainable production and consumption of resources, particularly in sub-Saharan Africa where effective governance and waste management systems are lacking. In this paper, we develop an empirical understanding of the influence of public governance system on promoting circular economy (CE) for plastic actions among individuals. Through a survey of 1475 participants across five sub-Saharan African countries, we tested five hypotheses drawing on New Governance Theory and CE Action Recipe to explore the relationship between governance and individual’s actions supporting CE for plastic. We found that a governance system that supports CE practices and exhibits governance efficiency is positively associated with individuals’ actions supporting CE for plastic. The awareness of government policies, laws and regulations, institutions, processes, and programmes have a significant impact on individuals’ engagement in plastic circularity practices. The paper’s theoretical and governance implications highlight the relevance of public governance in shaping action towards a CE for plastic at the individual level.

## Introduction

The challenges of plastic waste remain an obstacle to the attainment of several UN intersecting sustainable development goals (SDGs) (Schroeder et al. 2019; Ayeleru et al. 2020; Kan and Miller 2022). The issue of plastic waste management has international and local implications (Sebille et al. 2015; Tulashie et al. 2022). Geyer et al. (2017), for example, estimates that globally, over 8300 million metric tons (Mt) of virgin plastics have been produced to date. As of 2015, approximately 6,300 Mt of plastic waste had been generated, of which only about 9% was recycled, 12% was incinerated, and 79% was accumulated in landfills or the natural environment. The volume and longevity of plastic waste coupled with a lack of effective public governance and waste management systems make the problem even more complex in developing countries, particularly in sub-Saharan Africa (SSA) (Adebiyi-Abiola et al. 2019; Ncube et al. 2021; Debrah et al. 2022; Chiaroni et al. 2022). While current per capita plastic consumption levels are comparatively low in SSA, the anticipated growth in plastic consumption necessitates forward-looking planning to address the challenges of plastic waste (UNEP 2018).

In recognition of the negative impact of the linear economy of “take, make, dispose” there has been an increase in research on the transition to circular economy (CE) as a solution to the problem of plastic waste (Geissdoerfer et al. 2017; de Jesus and Mendonça 2018; Mayer et al. 2019; Lee et al. 2021; Cramer 2022). We define CE as the concept of a cyclical, closed-loop, regenerative system where resource input and waste, emissions and energy leakage are reduced, and redesign and reuse of products are prioritised (Murray et al. 2017; Kirchherr et al. 2017; Liu et al. 2018; Beheshti et al. 2023). For plastic waste, a circular system ensures that the value of products, materials and resources is maintained in the economy for as long as possible (Merli et al. 2018).

The analysis of how to govern the transformational change from the present, mainly linear economy to a CE is being pursued by two main approaches. The first and widely researched is the network governance approach which involves collaboration among stakeholders who are keen to contribute to transformational change (Chembessi et al. 2021; Cramer 2022; Foraste 2023). This approach to CE leverages the collective knowledge, resources, and capabilities of the network to drive change, promote innovation, and create shared value (Ddiba et al. 2020). But the network governance approach can be constrained by power dynamics within collaborative networks, lack of accountability and public participation, poor information sharing framework, and the inability to scale and replicate of networks (Joshi et al. 2019; Ayeleru et al. 2020; Rótolo et al. 2022). The second governance approach is the public governance approach to CE (Fitch**-**Roy et al. 2020; Wu et al. 2021; Mihai et al. 2022; Cramer 2022). The pubic governance approach represents the conventional role of government as the guardian of the common good (Cramer 2022; Hu et al. 2024). This approach applied to the CE for plastic aims to manage and regulate the production, use, and disposal of plastic materials through policies, regulations, and public institutions to promote sustainable consumption and production and reduce waste to a minimum The limitations of the public governance approach have been reported in the literature and include poor regulatory regimes, insufficient CE infrastructure, poor public awareness and participation, and limited market and financial barriers (Geng and Doberstein 2008). These limitations are more pronounced in weak public governance contexts such as those found in many SSA countries (Godfrey et al. 2021; Essien and Spocter 2023).

However, across the literature on both the network and public governance approaches, the emphasis is on how CE is applied in business and policy-making (Spekkink et al. 2022), and surprisingly, we noticed the absence of empirical research that addresses the interaction between CE governance and citizen’s individual actions particularly as it relates to issues of plastic waste. Even where indicative research exists, these only provide collective action perspectives in the context of organised citizen initiatives (Smith and Seyfang 2013; Smith et al. 2014; Hossain 2016, 2018), social innovation (Pel et al. 2016; van der Have and Rubalcaba 2016; Loorbach et al. 2020) and digital ecosystem in controlling plastic waste (Khatami et al. 2023). These studies do not explore in-depth how an individual’s CE actions can be shaped by public governance or what public governance aspects motivate CE behavioural changes within individuals in their daily lives (Isenhour and Reno 2019; Temesgen et al. 2021). By overlooking this critical building block of social considerations, CE research has been criticised for being overly optimistic regarding the capacity of society to integrate government policies (Calisto Friant et al. 2020).

Addressing the gap in literature on the intersection between public governance approaches and individual’s actions as consumers is particularly important for the CE for plastic for two reasons. First, large volumes of plastic waste emanate from individuals as consumers (Hage 2007; Geyer et al. 2017; Mallick et al. 2023). The transition from a linear model to a CE, particularly in weak public governance contexts such as those found in many SSA countries (Grant and Oteng-Ababio 2021), strongly depends on the conduct of individuals (Ncube et al. 2021), some of whom are indigenous and institutionalised within informal settings (Oduro et al. 2021; Essien and Spocter 2023). Broadly speaking, the effectiveness of CE strategies for waste management in general hinges on a symbiotic relationship between public governance and individual responsibility (van Weelden et al. 2016; Parajuly et al. 2020). Public governance approaches, including policy-making, regulation, and infrastructure development, provide the necessary framework for implementing CE practices (Kharola et al. 2022). However, these approaches must be complemented by active participation from individuals (e.g., exploring reuse options, selling second hand, returning instead of stockpiling, and recycling instead of wrongly discarding), who are the primary agents of consumption, waste generation and CE (van Weelden et al. 2016; Oteng-Ababio). Second, circularity of the plastic value chain is about ensuring circular flows of plastics resources by closing the loop between post-use and production (Chiaroni et al. 2022). To close this loop and prevent plastic waste and leakage into the environment, an individual’s actions need to be aligned with the concept of circularity, and this alignment happens in the context of public governance and regulatory initiatives (or lack thereof). Hence, we set out to address this knowledge gap and respond to the call in literature of the need to understand the enabling factors for the diffusion of CE (Kirchherr et al. 2017; Schr**ö**der et al. 2019; Chiaroni et al. 2022; Cader et al. 2023), particular the factors that motivate an individual’s action supporting a CE for plastic as they intersect with governance frameworks (Zink and Geyer 2017; Corvellec et al. 2022; Spekkink et al. 2022). We argue that even though our research is focused on plastic CE, understanding the dynamics of the relationship between individual’s CE actions and governance and regulatory initiatives (or lack thereof) has broader CE implications for waste management in general. This is because both a CE and waste management systems must integrate individual actions with governance structures to create a cohesive approach to resource utilisation and waste reduction (Salvia et al. 2021).

The aim of this paper is to explore the enabling factors for the diffusion of the governance of plastic CE, specifically, the focus is on understanding the motivations behind individuals’ engagement in plastic CE actions and examining how these motivations intersect with the context of public governance. Hence, we ask: *what public governance aspects motivate plastic CE practices and actions in individuals within weak public governance contexts such as those found in many SSA countries*? To address this question, we developed and tested 5 hypotheses using survey data from 1475 participants across 5 SSA countries. The focus of the SSA context is deliberate as many. Many SSA countries exhibit weak waste management and public governance structures (Sibanda et al. 2017; Salvia et al. 2021) despite the opportunities of propagating CE practices with economic benefits (Zapata Campos and Zapata 2013). Hence, this context presents unique challenges and opportunities for understanding how governance frameworks can influence individual actions towards plastic CE, and to uncover how individuals’ motivations and actions can contribute significantly to CE practices despite the lack of strong regulatory support. The high levels of plastic waste generation, and the environmental challenges from inadequate waste management infrastructure also makes the region an urgent area of study for CE transition strategies (Adebiyi-Abiola et al. 2019; Debrah et al. 2022; Chiaroni et al. 2022). The findings from SSA contexts can, therefore inform policy development within the region and in regions with similar governance challenges across the global south.

Our results show how CE governance systems including codified policies, laws, and regulations, shape individual behaviours and influence their adoption of plastic circularity practices. The ability of governance system to achieve its objectives in the most effective and resource-efficient manner also plays a critical role in promoting the overall plastic CE in SSA. Our results also suggest that as the Individual Factor (which includes concern about plastic waste, willingness to take action, and a sense of environmental responsibility) increases, so does the likelihood of an individual taking action towards CE for plastic. Hence, we develop an empirical understanding of the role of governance in promoting plastic CE actions at the micro individual level, with theoretical and governance implications.

The rest of the paper is structured as follows. Section 2 presents the theoretical framework and hypotheses of the paper. Section 3 presents the method whilst section 4 presents the results. Section 5 discusses the theoretical and governance implications and finally, section 6 discusses the conclusion and limitations of the paper.

## Theoretical Framework and Hypotheses Development

Two analytical approaches to the implementation of CE exist in literature. The first approach focuses on macro-level strategies (Yuan et al. 2006; Geng and Doberstein 2008). Here, CE is adopted at an administrative and policy level such as country, region, or city and follows a top-bottom approach to CE. The micro-level of analysis, on the other hand, shifts its focus toward governance of firms and consumers and the intersections with circular behaviours, and focuses on a bottom-up approach, with the aim of spreading sustainability practices and culture among all society stakeholders (Ghisellini et al. 2016; Pomponi and Moncaster 2017). The micro-level analysis also emphasises the need to acknowledge and create sustainable patterns of consumption and production through the propagation of transformational governance in society from institutions to businesses and consumers (Sauv**é** et al. 2016; Ghisellini et al. 2016).

According to Lynn et al., (2000 p.236), governance encompasses the “regimes of laws, administrative rules, judicial rulings, and practices that constrain, prescribe, and enable government activity, where such activity is broadly defined as the production and delivery of publicly supported goods and services.” The government’s role in public governance is not a constant but a variable, given the existence of governance models ranging from state-centric to more society-centred approaches (De la Mora-De la Mora 2023). The ‘old’ governance embodies the traditional notion of top-down steering by national governments, and emphasises the extent of control the government can exert over social and economic activities (Kjaer 2004). In contrast, the ‘new’ governance is characterised by increased interactions between the centre and society as underpinned by the new governance theory (Rhodes 1996)- often involving more self-steering within networks and individual actions. New governance inculcates the concept of responsibility- which is predominantly understood as a *post-subjectivist* view -on responsibility that understands individualisation (Shamir 2008; Soneryd and Uggla 2015). Here, institutional boundaries are blurred, and political processes socially and spatially diffuse (Lund 2006). In practical terms, this means that individuals, rather than solely institutions, have an important role in advancing CE practices. This role can manifest in various ways. Individuals as consumers can be encouraged to make conscious choices regarding product usage, recycling habits, and overall sustainable consumption (Van Dam 1996; Essien and Spocter 2023). They can contribute innovative solutions to enhance resource efficiency and reduce waste, and participate in community engagement, encouraging collaboration, and advocating for supportive policies that incentivise and promote circular practices.

From a post-subjective perspective, governance transcends conventional institutional structures in recognition of the agency of individuals (Ulibarri et al. 2023) as active contributors to the meaning-making process. It posits that the efficacy of CE governance hinges on the *meaning work* by individuals as actors (Benford and Snow 2000). Meaning work- also termed as signifying work or meaning construction- encompasses the process of shaping, sustaining, and challenging an individual’s comprehension of the world (Benford and Snow 2000). This involves attributing subjective meaning to objects, expressions, and actions, facilitating the intelligibility of one’s surroundings (Goffman 1974; Blomsma et al. 2023). In the context of CE, the notion of meaning work becomes relevant as it illustrates how an individual as an actor within a CE governance system is shaped by their understanding of sustainability and determines appropriate actions. Various factors contribute to the meaning-work establishing process, such as CE governance contexts, enactment, present cues, and the ability to permit reinterpretation (Weick 1995; Weick et al. 2005). Meaning work aims to organise these factors and the overall experience of the world into a coherent framework, and to determine which understandings guide actions. Ultimately, meaning work is not about revealing the objective reality but focuses on creating *‘action recipes’* by aligning past experiences, internalised concepts, and new stimuli to form a foundation for action (Blomsma et al. 2023).

**Fig. 1:**
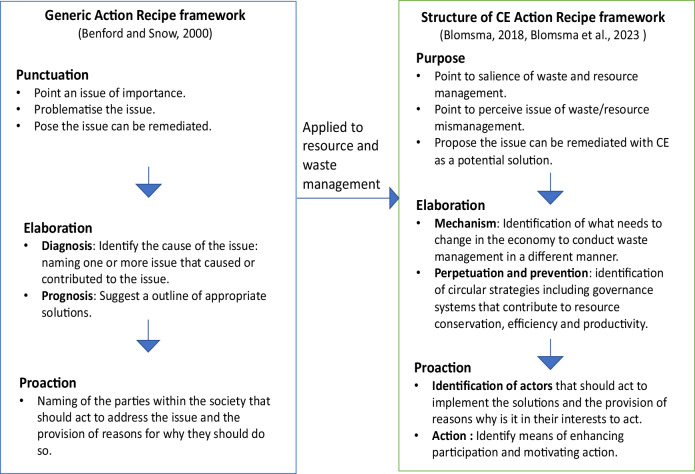
Theoretical conception of CE action recipe and individual action

In Fig. 1 below we build on the work of Blomsma (2018) and Blomsma et al. (2023) which is also an extension of the work of Benford and Snow (2000) by adding a CE ‘action’ element to the framework to make sense of how individuals’ behaviour intersects with plastic CE governance system and how such intersection may result in individual CE actions. At the defining a purpose stage of the action recipe framework, the focus is on the importance of effective waste and resource management, driven by the identification of problems (such as plastic pollution) or the absence of specific benefits (e.g., circularity and resource optimisation). Circular strategies are then formulated to address these issues. Subsequently, root causes are identified, linked to circular strategies, and relevant high-level actions are developed to mitigate the issue. This represents the elaboration step and can involve the establishment of a governance system that includes both codified, regulatory, and formal responsibilities, and those based instead on individual actions (Savini and Giezen 2020). Proaction involves the identification of actors necessary for implementing the proposed solution, along with motivations for their beneficial involvement (Blomsma et al. 2023). How these stages of CE action recipes are achieved is well documented in literature (see for example Blomsma 2018; Blomsma et al. 2023; Islam et al. 2023). But a micro level analysis is required to identify what aspects of public governance motivates action and means of ensuring individual participation in CE practices.

In the context of the public governance of CE for plastic, we argue that action happens when individuals participate in CE based on their understanding of the benefits of CE. This is triggered by cues and interpretation of self-motivation, including an individual’s sense of responsibility or concern for the environment, and the interpretation formal CE public governance system (or lack thereof) (Isenhour and Reno 2019). For example, the plastic and solid waste management governance system in Kenya, as outlined by the National Environmental Management Authority (NEMA), aims to promote the adoption of CE practices across the municipal authorities with the support of technology technologies (Salvia et al. 2021). But despite the plastic bag bans legislated in Kenya in 2017, plastic bags are ever-present in markets across the country. NEMA recognises the, challenge to achieving this in Kenya, including issues related to funding, governance, infrastructure, and factors related to individual’s ability to act. It advocates for governance system that establishes a common platform for action between stakeholders to overcome these challenges with a view of waste (particularly plastic waste) as a means of job creation and economic development (Moore 2012; Millington and Lawhon 2019). The solid waste management governance system in Zambia acknowledges CE issues related to indiscriminate dumping, overflowing landfills, and the limited circularity of waste streams, including plastic waste (Clube and Hazemba 2024). However, despite the integration of several CE practices in the public governance framework, the predominant approach in practice remains predominantly linear (Chileshe and Moonga 2019; Edomah 2020). Delays in progress can be attributed to policy obstacles, particularly evident in issues related to ownership and coordination, poor implementation and enforcement of current laws, lack of encouragement for innovations, and a failure to integrate the informal sector (Edomah 2020; Fitch**-**Roy et al. 2021; Clube and Hazemba 2024).

In Nigeria, a public governance system specific to plastic waste management is almost non-existent, despite the over 2.5 million tonnes of plastic waste generated annually and despite Nigeria’s ranking as the ninth globally among countries with the highest contributions to plastic pollution (Duru et al. 2019; Dumbili and Henderson 2020). The broader solid waste management approach in Nigeria is hinged on the monopoly of agencies of state governments (sub-national governments) who are constrained by their lack of capacity to tackle the problems of solid waste management. This is compounded by challenges associated with insufficient financial resources; non-compliance to laws and lack of individuals’ awareness and actions to mitigate plastic pollution (Ike et al. 2018). The governance challenges identified in Kenya, Zambia and Nigeria emerges as cues that constraint individual action and responsiveness to the CE benefits (Romeela and Thokozani 2015; Ezeudu et al. 2021; Olabomi 2024). In the case of Rwanda, governance of plastic CE been found to be more focussed and includes a formal governance system based on enacted laws, useful in mobilising the individual action and participation (Essien and Spocter 2023). In particular the Law N° 57/2008 of 10/09/2008 relating to the prohibition of manufacturing, importation, use and sale of polythene bags. This is matched with leadership and political commitments for the reduction of unnecessary plastic packaging and products. This commitment also motivates practices for individuals to participate in the work of government via individual action that helps in the propagation of public discourse and voluntary actions towards a CE for plastic (Ogutu et al. 2023). But even such governance approach found in Rwanda is not immune to the CE challenges encountered in the public sector such as financial constraints, limited human resources, poor infrastructure, poor supervision etc (Awortwi 2004; Spoann et al. 2019). Consequently, many scholars have increasingly called for research to mobilise individual action toward CE (Zink and Geyer 2017; Corvellec et al. 2022; Spekkink et al. 2022). Hence, our theoretical framework considers the motivation that emerges when citizens as individuals intersect with public governance system (Bingham et al. 2005). Indicative research shows that governance system influence participation and actions towards CE (Farla et al. 2012; Cramer 2022). We, therefore, hypothesise that a CE governance system will shape an individual’s ethos of modern society, which influences their actions towards CE. Thus, the following hypothesis (H1) was postulated:

*H1: There is a positive relationship between Governance System and individual’s action towards plastic CE*

Beyond the availability of a public governance system is the need for governance efficiency. Governance efficiency refers to the aspects of governance that affect the effectiveness of the aforementioned governance system (Christensen 2021; Cramer 2022). Governance efficiency affects the ability of government institutions to leverage their knowledge and power to effectively formulate, implement, and monitor regulations, engage stakeholders, and allocate resources towards plastic CE practices. (Georg 2015; Fratini and Jensen 2017; Fratini et al. 2019). Governance efficiency builds confidence in the public regarding the implementation of efficient plastic waste management programs upon which the transition to plastic CE can be achieved. It is also important for actively engaging with stakeholders, including businesses and consumers, to promote sustainable practices (Bellezoni et al. 2022).

Governance efficiency is important in the creation of an enabling environment for plastic CE by providing the necessary commitment, resources, funding, and support (Araujo Galvão et al. 2018; Ormazabal et al. 2018). It ensures that institutions shape the knowledge and ideas that circulate within society, influencing how individuals and organisations understand and approach waste management and the transition to CE (Fischer and Newig 2016; Kanda et al. 2019). Because governance efficiency is essential for the effective implementation of CE governance systems, we anticipate that it affects the overall efficiency of plastic CE. When CE governance is efficient, it can effectively lead to better enforcement of regulations, effective engagement with stakeholders, and efficient allocation of resources towards sustainable CE practices, which can lead to a more efficient overall plastic CE practices. We define the overall plastic CE efficiency as the effectiveness of plastics CE framework from the combined effects of governance system that ensures efficient collection, sorting, and recycling of plastic waste (Corona et al. 2019; Maione et al. 2022), and the individual’s concern about plastic waste in their environment, willingness to act, and whether an individual feels that they are responsible for the environment (Niskanen et al. 2020; Calisto Friant et al. 2020). Hence, we state the following two hypotheses (H2 and H3):

*H2: There is a positive relationship between Governance Efficiency and Governance System affecting plastic CE action*.

*H3: Governance efficiency positively influences the overall efficiency of CE for plastics*.

Circularity emerges as a theoretically, practically, and ideologically notion (Corvellec et al. 2022). Norms and discourses shape the dominant ideological beliefs, values, and attitudes towards CE and influence the diffusion of CE practices (Siyambalapitiya et al. 2018). In SSA, for instance, the norms of circularity are entrenched in the practicality and ideology of livelihoods of indigenous communities in the form of creating regenerative systems that sustain, restore, and are respectful of the Earth (Calisto Friant et al. 2020). An individual’s plastic CE action can, therefore, include one or several actions such as avoiding the use of single-use plastic items, reusing plastic items, and proper disposal (Valenzuela and Böhm 2017; Vonk 2018). Given the scale of the problem of plastic waste (Geyer et al. 2017), individuals are concerned about plastic waste in their environment and its impact on their health. In fact, a number of variables can be associated with an individual’s circularity action. As discussed earlier, this can include an individual’s concern about plastic waste in their environment, willingness to take action and engage in plastic CE, and whether an individual feels that they are responsible for the environment (Niskanen et al. 2020; Calisto Friant et al. 2020). We called these variables Individual Factor and propose the following hypothesis (H4):

*H4: There is a positive relationship between Individual Factor and an individual’s action supporting CE for plastics*.

Individual Factor can affect how governance efficiency impact individual’s plastic CE action (Niskanen et al. 2020; Calisto Friant et al. 2020). The way individuals think about, value, and prioritise circularity can influence their willingness and ability to adopt CE practices (Al-Awlaqi and Aamer 2022). For example, indicative research shows that individuals who have a strong environmental consciousness or a deep sense of responsibility towards the environment may be more likely to engage in circular practices (Calisto Friant et al. 2020), regardless of governance efficiency. Conversely, even when policies and regulations are in place, individuals who do not have a strong sustainability mindset may not be motivated to engage in circular practices. Therefore, we postulate hypothesis (H 5) as follows.

*H5: Individual Factor can mediate Governance Efficiency and Individual’s plastic CE action.*

## Research Method

### Framework Construct

**Fig. 2:**
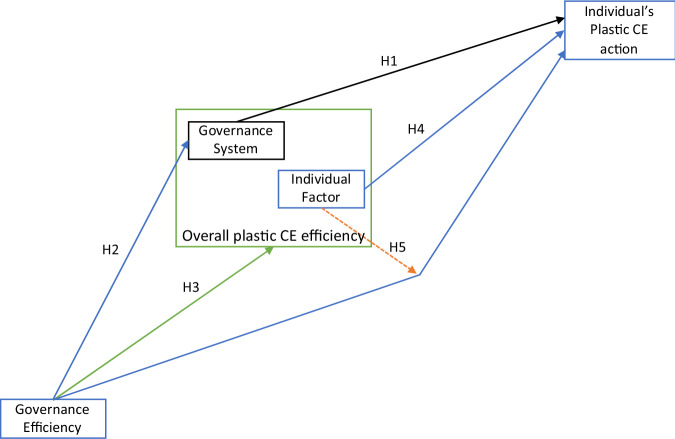
Conceptual framework and hypotheses

Our paper aims to provide empirical evidence of the enabling CE factors (Kirchherr et al. 2017; Chiaroni et al. 2022) that intersect public governance of CE and individual’s plastic CE actions (Zink and Geyer 2017; Isenhour and Reno 2019; Temesgen et al. 2021; Corvellec et al. 2022; Spekkink et al. 2022). Hence, we posit that two broad factors will directly determine an individual’s plastic CE action, i.e., the Individual Factor and the Governance System (Calisto Friant et al. 2020; Cramer 2022; Morseletto 2023; Van Opstal and Borms 2023). Both the Individual Factor and the Governance System are latent variables determined by a number of measurement variables. We illustrate the latent variables and our 5 hypotheses in the conceptual framework in Fig. 2 below.

Our construct measures an individual’s plastic CE action by their responses, i.e., if they have taken action towards plastic CE before. This forms our dependent variable with a binary outcome of “No” or “Yes”. The Individual Factor in our model is a latent variable with 4 indicators (INDF1 – INDF4). This factor assesses how concerned an individual is about plastic waste and how responsible an individual feel for the environment, both measured on a 5 Likert scale. The remaining 2 indicators under the individual factor control for individuals’ awareness of plastic waste laws in their respective countries and their willingness to take action. These are measured using binary outcome of “No” or “Yes”.

The Governance System is a latent variable with 3 indicators (GOVS1 – GOVS3). They are measured by the overall effect of plastic waste management (binary), the response to the question ascertaining if the barrier to action is related to the knowledge of CE laws, regulation and policy mandates, and the response to the question regarding the convenience of government collection infrastructure. Both questions are measured on a 5-point Likert scale. Finally, Governance Efficiency is made up of 5 indicators (GEFF1 – GEFF5). This mediator controls for the efficiency of CE in the country using responses to the question of CE efficiency, confidence in the country’s law enforcement, and political factors that incentivise plastic CE, all measured also on a scale of 1–5. The mediator also uses the indicator, asking if the government is doing enough with a binary outcome of “No” or “Yes”. Table 1 presents the snapshot of our factors, respective indicators and their corresponding survey question area. The table also presents the summary statistics of all variables in our model. Variables that are binary have a minimum value of 0 and a maximum value of 1 while other variables (Likert scale) have a minimum of 1 and a maximum of 5.

**Table 1:** Variables definition

Variables	Names	Survey Question areas	Mean	Std. dev.	Min	Max
Dependent Variable	ACT	Take action towards Plastic CE	0.6988	0.4589	0	1
Individual Factor (Latent Variable)	INDF1	Concerned about plastic waste	3.7648	1.0806	1	5
	INDF2	Aware of laws on CE	0.3372	0.4729	0	1
	INDF3	Willing to take action	0.9143	0.2800	0	1
	INDF4	Responsible for environment	4.1652	0.8398	1	5
Governance System (Latent Variable)	GOVS1	Available plastic CE programs	0.8430	0.3640	0	1
	GOVS2	Knowledge of laws, regulation and policy mandates	2.9272	1.3839	1	5
	GOVS3	Available govt collection	4.1101	0.7955	1	5
Governance Efficiency (Latent Variable)	GEFF1	Efficiency of waste management in country	3.0245	1.3179	1	5
	GEFF2	Visible government efforts	0.5187	0.4998	0	1
	GEFF3	Confidence in law enforcement	3.4310	1.1317	1	5
	GEFF4	Law, regulatory and policy that encourage efficient plastic CE	3.9959	0.9497	1	5
	GEFF5	If plastic collection mechanisms are convenient or not	0.7627	0.4255	0	1

**Table 2:** Statistical representation of collected data

Variable	Category	Frequency	Percent
	Female	730	49.49
Gender	Male	745	50.51
	Total	1,475	100
	18–24	348	23.64
	25–34	504	34.24
Age	35–44	350	23.78
	45–54	191	12.98
	55–64	61	4.14
	65–74	18	1.22
	Total	1472	100
	Primary	212	14.84
	Secondary	565	39.54
Education	Vocational training	132	9.24
	Tertiary	453	31.7
	Post-graduate	67	4.69
	Total	1,429	100
	Kenya	329	22.31
	Nigeria	429	29.08
Location	Namibia	88	5.97
	Rwanda	341	23.12
	Zambia	288	19.53
	Total	1475	100

### Data Collection

To test the hypotheses in our conceptual framework, we utilise Structural Equation Modelling (SEM) because it is a robust and powerful statistical tools used in CE research (Khan et al. 2020; Centobelli et al. 2021). We opted for a SEM as a quantitative approach because SEM allows us to analyse complex relationships between observed and latent variables, thereby providing a comprehensive understanding of the interactions within our conceptual framework (Hair et al. 2011). To achieve this, we surveyed 1475 individuals in Nigeria, Kenya, Zambia, Namibia, and Rwanda for this study because they cover a wide representation of SSA. Our choice of SSA as a geographical region is motivated by scale of the problem of plastic waste in the region and the varying levels of effectiveness of governance and waste management systems (Adebiyi-Abiola et al. 2019; Ncube et al. 2021; Debrah et al. 2022; Chiaroni et al. 2022). For instance, Nigeria, as the 9^th^ largest global plastic polluter, faces a considerable challenge with plastic waste, inadequate waste management infrastructure and governance systems (Ike et al. 2018; Ezeudu et al. 2021; Olabomi 2024). Kenya, is known for its progressive policies, but witnessed a failed comprehensive ban on plastic bags (Salvia et al. 2021). It, therefore, offers insights into the ineffectiveness of stringent regulations in governance context. Zambia provides a perspective from a landlocked country with unique logistical challenges in waste management amidst a rapid demographic shifts (Clube and Hazemba 2024). Namibia, with its low population density, presents a contrast in terms of waste generation and management practices (Kadhila et al. 2023; Erasmus et al. 2024). Lastly, Rwanda is recognised for its pioneering plastic band and effective enforcement, making it a model for policy implementation (Ogutu et al. 2023). Hence, by covering these five countries, we aim to capture a broad spectrum of experiences and practices in plastic waste management across SSA to ensure the generalisability and applicability of our emerging findings to other contexts similar to those within and beyond SSA.

Geographic diversity was ensured by a wide continental coverage (East, West and South); significant differences in economy size (Nigeria with a GDP of $375.8 billion versus Rwanda with a GDP of $9.137 billion) and population (190 million in Nigeria to 2.5 million in Namibia). A stratified random sampling technique (Kadilar and Cingi 2005) was used to select individuals across rural, peri-urban, and urban communities across the 5 sampled SSA countries to ensure representation and confidence in the collected data set. The strata were based on the living conditions (rural, peri-urban, and urban) and socioeconomic status. A door-to-door electronic structured questionnaire was designed and administered (between March - April 2021) based on the research hypotheses and literature review. The questionnaire was pretested and validated before the actual data collection. The data collection was conducted by trained field researchers who followed ethical and privacy protocols. We opted for a quantitative approach because it enables us to objectively measure and analyse the data collected from a large sample size, thereby ensuring the reliability and generalisability of our findings (Bowerman et al. 2011; Etikan et al. 2015). Quantitative methods facilitate the testing of hypotheses through statistical analysis (Field 2009). As opposed to qualitative methods, this allowed us to quantify the strength and direction of relationships between variables. Additionally, this approach provides the rigor needed to validate our conceptual framework and ensure that our conclusions are supported by empirical evidence (Khan et al. 2020; Centobelli et al. 2021).

Table 2 presents the summary statistics of observations in our data. The table shows the distribution by gender in which our sample is almost equally split between male and female. The distribution by age also shows that all age groups are represented, and the distribution implicitly reflects SSA demography where over 50% are below age of 35 (May and Turbat 2017). The distribution by education also shows that every group is represented with a fair distribution to reflect the demography in most SSA countries. The distribution by location shows that 4 countries (Kenya, Nigeria, Rwanda and Zambia) are well represented. However, Namibia still has about 6% of respondents in our data.

### Data Analysis

To analyse our dataset, we utilise SEM and opted for the covariance-based SEM (CB-SEM) (Hair et al. 2011) instead of the partial least squares SEM (PLS-SEM) because we are interested in understanding the relationship between the governance of plastic CE and actions of individuals towards plastic CE rather than attempting to predict the phenomena (Weston and Gore 2006). Hence, we test our hypotheses by assessing the significance of the regression coefficient for each of the factors in relation to the plastic CE action for H1, H2 and H4. For H3 and H5, we test the hypotheses by assessing the significance of their overall coefficients (post estimation tests). We reject the implicit null hypothesis whenever the P-value of the coefficient is less than 0.05, otherwise, we fail to reject the implicit null hypothesis when the P-value is greater than 0.05.

## Result

### Assessment of the measurement model

We allowed many of our variances to covary (which is one of the strengths of SEM) to satisfy the reliability of our indicator measurement. Table 3 presents other validity checks such as checking the Comparative Fit Index (CFI) of our model. A good model is expected to have a Comparative Fit Index (CFI) that is close to 1 (Kolade et al. 2022). Our measurement model gave 0.99 CFI. Furthermore, the CMIN/DF is expected to be less than 3 (Weston and Gore 2006; Kolade et al. 2022), our model returned 1.67.

**Table 3:** Post Estimation Tests: Fit Indices for both measurement model and structural model

Fit Indices	Measurement Model	Structural Model	Model Criteria
CMIN/DF	1.37	1.36	<3
CFI	0.99	0.99	>0.90

To assess the reliability of our indicators, we compared our model to the saturated model in Table 4. According to Anderson and Gerbing (1984) a good model should have a chi2 likelihood ratio greater than 0.05. In our case, the ratio was 0.085. Additionally, we compared the baseline model to the saturated model, where a good fit model should have a chi2 likelihood ratio of less than 0.05 (StataCorp, 2021). Our model reported a ratio of 0.000. Finally, our baseline comparison uses the Tucker-Lewis Index (TLI) which is a fit index commonly used in SEM to evaluate the goodness of fit of our model. TLI is also known as the Non-Normed Fit Index (NNFI). It is a relative fit index that compares the fit of the specified model to a null model (a model where all variables are uncorrelated. TLI close to 1 suggests a good fit (Hu and Bentler 1998, 1999). Our model produced a TLI value of 0.98. We therefore conclude that our model’s goodness of fit is good.

**Table 4:** Fit statistics

Fit statistic	Value	Description
Likelihood ratio		
chi2_ms(30)	41.109	model vs. saturated
p > chi2	0.085	
chi2_bs(78)	2062.526	baseline vs. saturated
p > chi2	0.000	
Population error		
RMSEA	0.016	Root mean squared error of approximation
90% CI, lower bound	0.000	
upper bound	0.027	
pclose	1	Probability RMSEA < = 0.05
Baseline comparison		
CFI	0.994	Comparative fit index
TLI	0.985	Tucker–Lewis index

### Structural model and hypothesis testing

After evaluating the goodness-of-fit indexes, we tested the research hypotheses describing the causal relationships among the constructs of the structural equation model. We accept the hypotheses with positive coefficient (β) and a p-value of less than 5% or lower. A p-value of 5% or lower is often considered to be statistically significant (Greenland et al. 2016). The result of the hypotheses testing is shown in Tables 5 and 6 show the summary of the hypotheses and their corresponding decision. H1 is positive and significant (β = 0.5470, *p* < 0.05). This means that Governance System is positively associated with plastic CE action of individuals. The implication of the result suggests that where there exists visible plastic CE government policies, laws and regulations, institutions, processes and programs, there is a significant impact on an individual’s adoption of circularity practices.

**Table 5:** Results of the structural equation model

	Coefficient	std. err.	z	*P* > |z|	[95% conf. interval]	
**Path- Latent variables**						
**GovSystem ->** **ACT**	0.5470954	0.2055346	2.66	0.008	0.1442549	0.9499358
**GovEfficiency ->** **GovSystem**	0.0769617	0.0391329	1.97	0.049	0.0002627	0.1536607
**GovSystem ->** **Overall effect**	0.930524	0.198721	4.68	0	0.5410379	1.32001
**IndividualF ->** **ACT**	1	(constrained)				
**GovEfficiency ->** **IndFactor**	−0.0174623	0.0143936	−1.21	0.225	−0.0456732	0.0107486
**Path- Observed variables**						
**IndividualF**						
**INDF1**	5.386597	1.932454	2.79	0.005	1.599056	9.174138
**INDF2**	1.505713	0.5101532	2.95	0.003	0.5058316	2.505595
**INDF3**	1.031595	0.3341967	3.09	0.002	0.3765818	1.686609
**INDF4**	3.705264	1.24525	2.98	0.003	1.264619	6.145909
**GovSystem**						
**GOVS1**	0.897627	0.1531306	5.86	0	0.5974964	1.197757
**GOVS2**	−0.7616968	0.2854602	−2.67	0.008	−1.321188	−0.2022052
**GovEfficiency**						
**GEFF1**	0.6148157	0.1135802	5.41	0	0.3922027	0.8374288
**GEFF2**	0.2343643	0.0499817	4.69	0	0.136402	0.3323266
**GEFF3**	1	(constrained)				
**GEFF4**	0.2298539	0.1064847	2.16	0.031	0.0211477	0.4385601
**GEFF5**	0.1263714	0.058552	2.16	0.031	0.0116116	0.2411312

**Table 6:** Summary of the hypotheses and decision

	Hypotheses	Path Coefficient β	*P*-value	Decision
${H}_{1}$	*There is a positive relationship between Governance System and individual’s action towards plastic CE*	0.5470	0.008	Positive and significant at 5%
${H}_{2}$	*There is a positive relationship between Governance Efficiency and Governance System affecting plastic CE action*.	0.0769	0.049	Positive and significant at 5%
${H}_{3}$	*Governance efficiency positively influences the overall efficiency of CE for plastics*	0.0691	0.039	Positive and significant at 5%
${H}_{4}$	*There is a positive relationship between Individual Factor and an individual’s action supporting CE for plastics*.	1.0316	0.002	Positive and significant at 5%
${H}_{5}$	*Individual Factor can mediate Governance Efficiency and Individual’s plastic CE action*	0.0246	0.359	Positive but not statistically significant.

In H2 and H3, we differentiate the availability of plastic CE Governance System from Governance Efficiency and how the Governance Efficiency affect the overall plastic CE efficiency. Given that resource availability has been identified as one of the prominent barriers of propagating CE Governance System in SSA, the focus on efficiency is needed to ensure that scarce resource are effectively used in the implementation of governance system. Our analysis shows that Governance Efficiency is positively associated with the Governance System and it is statistically significant (*β* = 0.0769, *p* < 0.05), therefore, H2 is upheld. H3 is also upheld (β = 0.0691, *p* < 0.05) and shows that efficient governance practices play a critical role in promoting the overall plastic CE in SSA.

H4 was upheld and confirms that there is a positive relationship between Individual Factor and individual’s plastic CE action (β = 1.0316, *p* < 0.05). This finding brings to focus the role of individuals as consumers and users and shows that individuals are willing to take self-motivated CE action.

H5 posed that individual factor, as a mediator, could influence the relationship between governance efficiency and an individual’s plastic CE action. Our findings show that while the correlation between governance efficiency and an individual’s plastic CE action is positive (β = 0.0246), the p-value of 0.4 makes the result statistically insignificant.

## Discussion

### Theoretical Implications

We explore the diffusion of the CE for plastic, specifically, focusing on understanding the enablers of individual action and participation in plastic CE within the context of public governance in SSA. We draw on action recipe (Benford and Snow 2000; Blomsma 2018) and theorised new governance framework from a *post-subjectivist* view (Shamir 2008; Soneryd and Uggla 2015) to make sense of the important role individuals have in advancing CE practices. Our findings provide a refine understanding of extant plastic CE governance theory as it provides a micro level analysis of individuals as plastic CE actors within public governance context, closing the gap in CE literature which overlooks this dimension. This is important as plastic pollution, particularly in SSA, mostly emanate from individuals as consumers (Hage 2007; Geyer et al. 2017; Mallick et al. 2023), and limited availability of resource in SSA context constraints the operation of formal waste management systems (Godfrey et al. 2021). Hence CE public governance that motivates actions from citizens as individual consumers becomes very relevant in this context (Geng and Doberstein 2008; Ghisellini et al. 2016; Pomponi and Moncaster 2017).

From the results of H1, H2 and H3, our work highlights how CE governance systems including codified policies, laws, and regulations, can shape individual action and influence their adoption of plastic CE practices. The ability of governance system to achieve its objectives in the most effective and resource-efficient manner possible also plays a critical role in promoting the overall plastic CE efficiency in SSA. Efficient governance system would aim to reduce plastic waste and promote the reuse, recycling, and recovery of plastic materials through effective waste management processes, the enactment of policies that are effective and build confidence in individuals, and support the economic benefits of waste (Geng and Doberstein 2008; Millington and Lawhon 2019; Godfrey et al. 2021; Maione et al. 2022). Hence, beyond the existence of governance system, our findings show that an efficient governance system also motivates individual action towards plastic CE. This findings align with the literature focusing on CE in businesses which suggest that a governance system that support the transition to CE, motivate innovations that addresses changes and develop enabling political agendas (Farla et al. 2012; Debrah et al. 2022). For instance, as noted in the case of Rwanda, efficient governance system ensures citizen participate in community service clean-up activities known as Umuganda. This reinforce the importance of individual responsibility for waste management (Ogutu et al. 2023; Essien and Spocter 2023). In this sense, our study shows the nuanced perspective of how efficient governance system can be consider as a form of cues that facilitates (re)interpretation and establishes meaning work. The cue can emerge from the an individuals’ visibility of how government institutions utilise knowledge and power to effectively formulate, implement, and monitor regulations and engage stakeholders in the propagation of plastic CE practices. (Georg 2015; Fratini and Jensen 2017; Fratini et al. 2019). This influences individual action and shapes subjectivity towards plastic CE actions. Our research indicates a crucial link between individuals’ perceptions of the governance system’s efficiency and the effectiveness of their actions within the context of plastic CE. This connection highlights a fundamental aspect of the action element of the CE action recipe we introduced in theorising our research (Benford and Snow 2000; Blomsma 2018; Blomsma et al. 2023). It suggests that individuals are not passive bystanders subject to governance; rather, they actively engage in shaping and reinforcing governance systems (Liu et al. 2009; Cramer 2022). Once CE purpose is identified, and governance system established at the elaborative stage of the action recipe, our findings shows that the degree to which actors perceive the governance system’s effectiveness significantly influences the outcomes of their actions to participate in plastic CE (Lund 2006). This is useful in mobilising the action of individuals in informal setting in recognition of their supplementary CE practices and the impact they can make to waste management given the challenges faced by both private and public sector within the SSA context (Essien and Spocter 2023).

In H4 we test the relationship between Individual Factor and an individual’s plastic CE action. The result suggests that as the Individual Factor (which includes concern about plastic waste, willingness to take action, and a sense of environmental responsibility) increases, so does the likelihood of an individual taking action towards plastic CE. This action could include avoiding single-use plastic items, reusing plastic items, and proper disposal (Corona et al. 2019; Maione et al. 2022; Shah and Yang 2023). The Individual Factor essentially forms the ingredients of ‘action’ in the action recipe for plastic CE (Blomsma 2018; Blomsma et al. 2023). The concern about plastic waste in the environment can be seen as the motivation or the ‘why’ of the action. The willingness to take action and engage in plastic CE can be seen as the commitment or the ‘will’ of the action (Benford and Snow 2000; Blomsma 2018). The feeling of responsibility for the environment can be seen as the ‘moral or ethical’ grounding of the action. The actions themselves - avoiding single-use plastic items, reusing plastic items, and proper disposal - are the steps of the action recipe (Maione et al. 2022; Shah and Yang 2023). They are the practical manifestations of the Individual Factor. The positive significance of this hypothesis suggests that our theoretical conception of the action recipe can be effective when individuals are concerned, willing, and feel responsible. This finding expands the literature of environmental value and shows the importance of an individual’s concern, will, and morals on pro-environmental actions (see for example Nilsson et al. 2004; Shah and Yang 2023; Steg et al. 2011).

H5 add an important dimension to the action recipe argument. While the action recipe focuses on concern, will, and morals of individuals (the Individual Factor), H5 highlights the role of broader social and institutional contexts (Governance Efficiency) in influencing these factors (Ranta et al. 2018). Although the correlation between governance efficiency and an individual’s plastic CE action is positive, the statistical insignificance (p-value of 0.4) suggests that governance efficiency alone may not be a strong determinant of an individual’s plastic CE action. This result shows the complexity of the issue - individual actions towards plastic CE are influenced not only by personal attitudes and behaviours but also by the efficiency of governance systems and other external factors. This could mean that the effectiveness of the action recipe is contingent on the broader context within which it is applied. For instance, an individual might be willing and motivated to take action (e.g., avoiding single-use plastic items, reusing plastic items, and proper disposal), but if the governance system does not provide the necessary infrastructure or incentives, the individual might find it difficult to carry out these actions. Therefore, efforts to drive plastic CE practices must consider both the Individual Factor (concern, will, and morals) and the facilitating environment (governance efficiency and broader social and institutional contexts) (Schr**ö**der et al. 2023). By improving governance efficiency and addressing other external factors that influence action, it may be possible to create a more conducive environment for individuals to follow the action recipe. This could lead to more significant and sustained positive changes in plastic CE actions (Wang et al. 2018; Christensen 2021).

### Policy Implications

In practice, our work provides important findings that can be used by policymakers for governance reforms in SSA to develop policies, laws, and regulations that support the transition to a CE for plastics. Our findings emphasise the need for an efficient CE governance system that aims to reduce plastic waste and promote reuse, recycling, and recovery of plastic materials. This can be achieved through effective waste management processes, enactment of policies that are effective and build confidence in individuals, with visible impact, and support for innovative plastic recovery processes (Georg 2015; Fratini and Jensen 2017; Fratini et al. 2019; Christensen 2021; Cramer 2022). But the challenge to establish an efficient governance system must be noted, particularly in the SSA context. Resource constraints and limitations in institutional capacity can all impose restrictions on the efficiency of governance systems (Awortwi 2004; Spoann et al. 2019). Therefore, it becomes crucial to tailor solutions that consider the particular ways in which CE action recipe are employed to validate the governance system(s) enacted for plastic CE. Additionally, our study show that the efficiency of governance systems alone is insufficient in addressing SSA’s plastic waste challenges, hence the need to pay attention to individual factors that motivate plastic CE. To achieve this, policymakers should leverage the concerns of individuals about plastic waste in their environment, their willingness to take circularity actions, and their sense of responsibility towards the environment, to shape policy discourse in favour of plastic CE (Valenzuela and Böhm 2017; Vonk 2018).

### Conclusion and Future Research Directions

This empirical study finds that a governance system that supports CE practices and exhibits efficiency is positively associated with individuals’ adoption of actions supporting CE of plastic. Awareness of plastic CE government policies, laws, regulations, institutions, processes, and programs has a significant impact on individuals’ engagement in circularity practices. The research also shows that the Individual Factor - which includes concern about plastic waste, willingness to take action, and a sense of environmental responsibility- is positive associated with an individual’s plastic CE actions. The research highlights the relevance of public governance in shaping action towards plastic CE at the individual level. The study’s theoretical and governance implications can inform policymakers and practitioners in developing effective governance strategies and policies to drive circularity practices and address plastic waste challenges. This is particularly useful in the SSA context where resource availability has been identified as one of the prominent barriers to propagating CE.

Finally, the limitations of the study should be noted. While we mitigated some of the limitations of the door-to-door survey by providing rigorous training for data collectors and implementing a robust electronic data collection platform with geolocation, it is still possible that respondents may have provided answers they believe the interviewer wanted to hear. Nonetheless, we believe that this data collection method is best suited for collecting survey data from participants with varied levels of education involved in a technical research topic. Also, while the study provides valuable insights on the impact of public governance in shaping action towards plastic CE, this is mainly through the examination of the relationships between governance system, governance efficiency, and individual factors. It does not account for other potential factors that could also play a role, such as economic factors, technological capabilities, or social norms. Future research could consider a more comprehensive analysis that includes these additional factors. Future research should also be conducted to draw on aspects related to the plastic CE behavioural disposition including tendencies or inclinations of individuals to engage in behaviours that support the principles of plastic CE within SSA. Such research might benefit from qualitative methods that can explore in-depth aspects of an individuals planned behaviour to compliment the action recipe perspective of this paper.

## Data Availability

Survey data will be made available on request.
